# The Comparative Function of Each Kidney
**Handbook of the Surgery of the Kidneys.* By W. Bruce Clarke. "Oxford Medical Manuals." London: Henry Frowde and Hodder and Stoughton. 1911. Pp. 199.


**Published:** 1911-07-01

**Authors:** 


### SURGERY.

### THE COMPARATIVE FUNCTION OF EACH KIDNEY.*

The great advances that have been made in urinary surgery of late years have been made possible by the improved methods of examination which are now employed. The ultimate aim of this examination is to gain a knowledge of the functional activity of each kidney separately. Older practitioners may say that such things were not done in their day and the patients seemed to do all right; but they forget that even then some rough tests for telling the renal function were in vogue. This was frequently limited to an estimation of the total urea; at other times an abdominal exploration was first made and the other kidney palpated before the damaged one was touched. The more risky abdominal nephrectomy had then to be performed, or else a separate incision made in the lumbar region. With these means many conditions now amenable to surgery were left alone, and the mortality of those that were touched was higher than at the present day.

### The Functional Activity of the Kidney.

When an operation on a kidney is deemed advisable, the functional activity of the other kidney should be learnt because it may be absent under two conditions. First, the organ may be non-existent; and secondly, it may be already destroyed by disease. The functional activity of the second kidney may be estimated either with the aptoscope or by collecting the urine from each side separately.

In the former case, it may be sufficient to see the urine coming down the ureter; if none comes down the diseased side and yet the total amount of urea passed per diem is high, then it may be assumed that all comes from the sound side and that this kidney is already doing all the work. The examination is more complete if some coloured substance, such as methylene blue, be injected into the muscles. This should appear in the urine in fifteen minutes, and if it be seen coming down the sound side in this time, the kidney may again be considered to be efficient in carrying on the excretory functions of the body.

### Segregation.

In other cases the urine must be collected separately from the two sides. This can be done by means of the segregation, whereby the bladder is divided into two halves, or can be done with greater accuracy by passing a catheter by means of a cystoscope into each ureter separately. This latter must be avoided, however, when there is any inflammation of the bladder, for fear of infecting the kidneys. As a matter of fact, the number of times in which the examination is necessary in the presence of a cystitis is not very great, and so this criticism does not impair the value of the method.

By these means the amount of urine passed by the two kidneys in the same time can be discovered. The absence of albumen in the urine from one side can be proved or disproved, and the amount of urea

*Handbook of the Surgery of the Kidneys.* By W. Bruce Clarke. "Oxford Medical Manuals." London: Henry Frowde and Hodder and Stoughton. 1911. Pp. 199.

passed by the two organs can be compared; if anything further be necessary, phoridzin can be injected subcutaneously. The normal kidney should excrete this substance as sugar, but if the organ be at fault it will not do this.

The exact details of such an examination need only be learnt by the specialist, but it is necessary for the general practitioner to know the principles of this and other parts of urinary surgery. Greater faith is often placed in him by the patient than in the surgeon, and he must be in a position to advise the patient to rely on the specialist's knowledge. Mr. Bruce Clarke's little book on the kidneys will be found most valuable for such a purpose. The main principles of renal surgery are excellently described without too much troubling with details.

